# Regional brain extracellular markers of cerebral ischaemia after aneurysmal subarachnoid haemorrhage: a combined cerebral microdialysis and perfusion CT study

**DOI:** 10.1186/2197-425X-3-S1-A814

**Published:** 2015-10-01

**Authors:** C Patet, T Suys, H Quintard, J-B Zerlauth, M Oddo

**Affiliations:** Neuroscience Critical Care Research Group - Intensive Care Medicine, CHUV - Lausanne University Hospital, Lausanne, Switzerland; Anesthesia and Critical Care, Nice University Hospital, Nice, France; CHUV - Lausanne University Hospital, Radiology, Lausanne, Switzerland

## Introduction and Objectives

Detection of cerebral ischemia after aneurysmal subarachnoid hemorrhage (SAH) remains challenging, particularly in comatose patients. The aim of this study was to examine the value of cerebral microdialysis (CMD) to predict cerebral ischemia, diagnosed by perfusion CT (PCT) imaging.

## Methods

We analyzed 48 PCT from 20 SAH patients (age 59 ± 8 years, median WFNS 4 [interquartile range 3-5]) monitored with CMD (in apparently normal brain) as part of standard care. PCT was categorized as *ischemic* (cerebral blood flow [CBF] < 32.5 mL/100g/min with a mean transit time >5.7 sec) vs. *non-ischemic*. Cerebral extracellular levels of lactate/pyruvate ratio (LPR) >40 with glucose < 1.0 mmol/L were used as thresholds for brain tissue ischemia (BTI).

## Results

**Figure 1:**
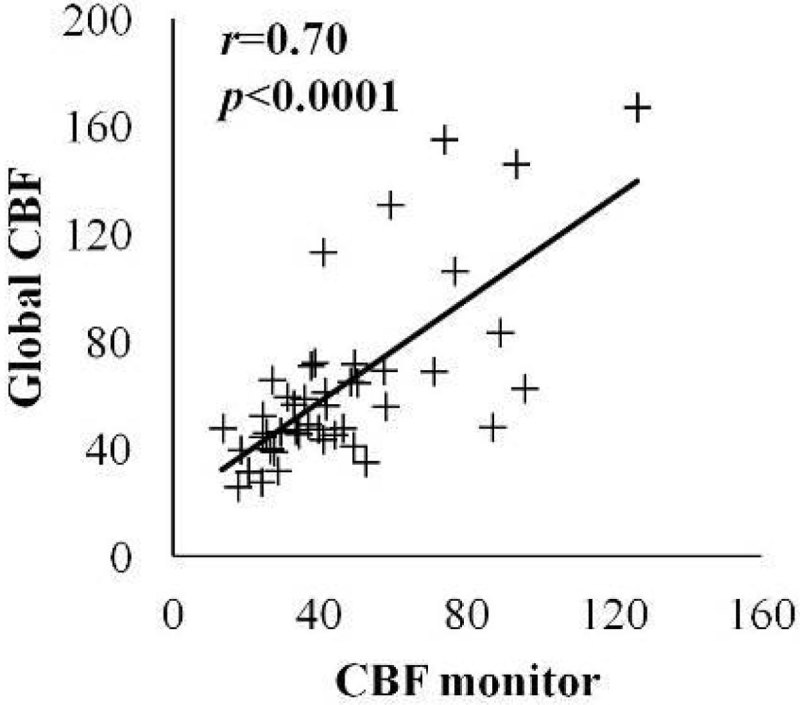
**Regional and global PCT data correlation.**

Regional CBF (around the CMD probe) correlated significantly with global CBF (averaged from bilateral anterior and middle cerebral arteries; Pearson's *r* = 0.70, *p* < 0.0001; Figure 1).

**Figure 2:**
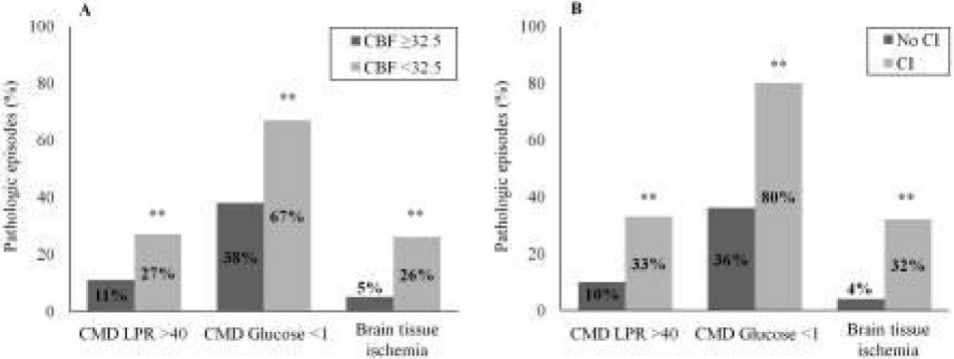
**CMD pathologic samples according to PCT.**

Ischemic PCT (n = 13; 10 patients) showed higher CMD LPR (48 ± 37 vs. 30 ± 10 in non-ischemic PCT) and lower CMD glucose (0.9 ± 0.8 vs. 1.4 ± 0.8 mmol/L; both *p* < 0.001). BTI was more frequent in ischemic PCT (32% vs. 4%, *p* < 0.0001; Figure 2) and correlated significantly with cerebral ischemia on PCT (correlation coefficient 2.72 [95% confidence interval 1.11-6.63], *p* = 0.028; generalized estimated equations analysis). A CMD pattern of BTI had a 72% positive predicted value and an 81% negative predictive value for detecting cerebral ischemia on PCT.

## Conclusions

Cerebral microdialysis appears accurate to detect cerebral ischemia at the bedside in comatose SAH patients and may be a valid complementary neuromonitoring tool in this setting.

## Grant acknowledgment

Supported by Grants from the Swiss National Science Foundation and The Novartis Foundation for Biomedical Research.

